# Wound trauma mediated inflammatory signaling attenuates a tissue regenerative response in MRL/MpJ mice

**DOI:** 10.1186/1476-9255-7-25

**Published:** 2010-05-25

**Authors:** Stephen R Zins, Mihret F Amare, Khairul Anam, Eric A Elster, Thomas A Davis

**Affiliations:** 1 Regenerative Medicine Department, Operational and Undersea Medicine Directorate at the Naval Medical Research Center Silver Spring, MD 20910-7500, USA; 2 Department of Surgery, Uniformed Services University of the Health Sciences, Bethesda, MD 20814, USA

## Abstract

**Background:**

Severe trauma can induce pathophysiological responses that have marked inflammatory components. The development of systemic inflammation following severe thermal injury has been implicated in immune dysfunction, delayed wound healing, multi-system organ failure and increased mortality.

**Methods:**

In this study, we examined the impact of thermal injury-induced systemic inflammation on the healing response of a secondary wound in the MRL/MpJ mouse model, which was anatomically remote from the primary site of trauma, a wound that typically undergoes scarless healing in this specific strain. Ear-hole wounds in MRL/MpJ mice have previously displayed accelerated healing and tissue regeneration in the absence of a secondary insult.

**Results:**

Severe thermal injury in addition to distal ear-hole wounds induced marked local and systemic inflammatory responses in the lungs and significantly augmented the expression of inflammatory mediators in the ear tissue. By day 14, 61% of the ear-hole wounds from thermally injured mice demonstrated extensive inflammation with marked inflammatory cell infiltration, extensive ulceration, and various level of necrosis to the point where a large percentage (38%) had to be euthanized early during the study due to extensive necrosis, inflammation and ear deformation. By day 35, ear-hole wounds in mice not subjected to thermal injury were completely closed, while the ear-hole wounds in thermally injured mice exhibited less inflammation and necrosis and only closed partially (62%). Thermal injury resulted in marked increases in serum levels of IL-6, TNFα, KC (CXCL1), and MIP-2α (CXCL2). Interestingly, attenuated early ear wound healing in the thermally injured mouse resulted in incomplete tissue regeneration in addition to a marked inflammatory response, as evidenced by the histological appearance of the wound and increased transcription of potent inflammatory mediators.

**Conclusion:**

These findings suggest that the observed systemic inflammatory response of a severe thermal injury undoubtedly has an adverse effect on wound healing and tissue regeneration.

## Background

Wound healing is a complex process involving many cell types and mediators that regulate tissue repair. Successful wound healing and tissue regeneration depends on tightly regulated hemostasis, inflammation, matrix synthesis, proliferation, wound contraction and tissue remodeling to restore tissue function and integrity [1,2]. A thermal injury is among the most severe forms of trauma with effects both locally and systemically [3]. Patients with large burn injuries have a multitude of immunological alterations and impaired functions of multiple effector cells of innate immunity and acquired immunity (including macrophages, dendritic cells (DC), natural killer (NK) cells, and T cells) at the wound site or a systemic change in circulating inflammatory mediators [3-8].

Systemic inflammation can lead to profound suppression of the innate and adaptive immune system [4-8] resulting in increased sepsis, wound healing complications, multi-system organ failure, and remote organ injury at sites such as the lung, liver, small intestines, and brain, representing major causes of morbidity and mortality in burn trauma patients [3,9]. These thermally induced organ injuries appear to be caused by toxic inflammatory mediators produced by infiltrating activated neutrophils early after thermal injury that are associated with increased chemokine levels [10-13].

The complex balance between innate and adaptive immune cell function after a severe injury is vital in determining wound healing outcome [4]. Innate immune cells show a progressive increase in the production of pro-inflammatory immune regulatory molecules (IL-1β, IL-6, TNFα and PGE_2_), while cells of the adaptive immune system display counter-inflammatory responses such as IL-10 and TGFβ [13-15]. The interplay between pro- and anti-inflammatory mechanisms is key for avoiding further tissue damage beyond that of the primary insult and a systemic inflammatory response [4,6-8,13,16].

Mice of the MRL/MpJ strain have been reported to have a unique capacity for limited regenerative wound healing, as shown by the complete closure of 2-mm ear-hole wounds [17-19]. Excised tissue is quickly replaced with normal tissue architecture that retains its full functionality. In contrast, others have shown that small, open, excisional cutaneous wounds in MRL/MpJ mice heal with marked scarring and no evidence of tissue regeneration [17,20-22]. Recently, our laboratory reported that a severe thermal wound on the dorsum of MRL/MpJ mice heal with scar formation and a delay in two critical wound healing events: wound closure and myofibroblast development [22]. The mechanism(s) involved are unclear, but it appears that the anatomical site of the injury, the severity of the injury, and the milieu of pro- or anti-inflammatory cytokines are all critical factors in determining whether a wound heals with or without a scar [20,21,23-25]. Moreover, in the MRL/MpJ mouse model we have demonstrated that the systemic response to a severe thermal injury can trigger a lethal autoimmune response within weeks-to-months following severe traumatic injury [26]. Understanding the dichotomous role of innate immune responses and inflammation on tissue regeneration versus delayed healing and scar formation may ultimately lead to innovative approaches for treatment of severe wounds to promote accelerated and scarless healing as well as tissue regeneration.

In this study we show in wild-type MRL/MpJ mice that scarless ear-hole healing does not occur following a severe thermal injury at an anatomically remote site. During the early inflammatory phases of healing, we observed marked pathological cutaneous skin lesions on the ear pinnae, including hyperkeratosis, acanthosis, mononuclear cell infiltration and necrosis in close proximity to ear-hole wound margins. In addition, we observed a significantly augmented inflammatory response in the serum, lung, ear wound, and burn wound margin tissue by analyzing various chemokine/cytokine expression levels. These findings underscore the profound importance of the systemic inflammatory response following peripheral tissue injury which can modulate other cellular events critical in wound healing, as evidenced by the impediment of an otherwise normal and complete wound healing-tissue regenerative response in MRL/MpJ mice.

## Methods

### Animals

Age matched (8-12 weeks) female MRL/MpJ mice were purchased from the Jackson Laboratory (Bar Harbor, ME) and housed at the Walter Reed Army Institute of Research/Naval Medical Research Center (WRAIR/NMRC) animal facility, which is certified by the Association for the Assessment and Accreditation of Laboratory Animal Care International. All procedures were conducted using facilities and protocols approved by the Animal Care and Use Committee of WRAIR (protocols #K06-05 and K01-08). The experiments reported herein were conducted in compliance with the Animal welfare Act and in accordance with the principles set forth in the current edition of the *Guide for Care and Use of Laboratory Animals*, Institute for Laboratory Animal Resources, National Research Council, National Academy Press, 1996. Animals were housed 5 to a cage until study initiation, and individually housed thereafter using standard micro-isolator polycarbonate caging. Animal rooms were kept at 21 ± 2°C with 50 ± 10% humidity on a 12-hr light/dark cycle. Commercial rodent ration (Harlan Teklad Rodent Diet 8604) was available freely, as was acidified (pH = 2.5) water to control opportunistic infections.

### Burn wound model

Mice were anesthetized with an intraperitoneal injection of ketamine (75 mg/kg), xylazine (15 mg/kg), and acepromazine (2.5 mg/kg). After shaving the dorsum, the exposed skin was washed gently with room temperature sterile water and prepped with Betadine (a 10% povidone-iodine solution for skin disinfection). The Betadine solution from the prep area was wiped off using 3 series of sponge gauzes containing 70% isopropyl alcohol. A single full thickness circular burn (15 mm diameter; ~15% total body) was introduced with electrocautery Bovie (370-400°C for 1.5 sec; Bovie Aaron Medical, St. Petersburg, FL) on the mid-dorsum of each mouse. This protocol produces a histologically proven well demarcated, full thickness, anesthetic injury that is non-lethal (< 0.5% mortality). Wounds were treated with bacitracin (Pharmaderm, Melville, NY) (applied topically) immediately after wounding, left uncovered, and allowed to desiccate. Once mice recovered from anesthesia, they were placed alone in separate cages and maintained under standard conditions in the animal facility (as described above). Buprenorphine (Reckitt Benckiser Pharmaceuticals, Richmond, VA) 0.1 mg/kg SC BID was given on post-operative days 1 through 3 for pain management. No additional therapeutic intervention was provided, as administration of anti-inflammatory or analgesic drugs may introduce complications into the assessment of inflammatory responses. Wounds became covered with inflammatory eschar, and no infection was evident macroscopically. At various time points post injury, cohorts of mice were killed by carbon dioxide inhalation and subsequent cervical dislocation. At the time of killing, cardiac blood was collected and serum was obtained and aliquoted and stored at -80°C until use. Lungs and wound edge/margin tissue from the ear and skin were collected and stored in RNALater (Ambion, Austin, TX). All samples were kept at 4°C until use.

### Ear wound and ear-hole-closure measurements

A 2-mm through-and-through circular hole was made in the center of the cartilaginous part of each ear using a standard surgical biopsy punch (Acuderm, Inc, Ft. Lauderdale, FL) immediately following thermal injury. Ear-hole diameter size was determined using a 7X Bausch & Lomb ocular magnifier (Fisher Scientific) immediately following the wounding procedure (day 0) and at frequent intervals over a 31-day evaluation period. Mice with ear-holes that were not cleanly cut or altered in configuration by the mice through scratching within a few days of injury were excluded from the study. Typically, in non-injured mice, the circular wounds healed in a circular fashion whereby the mean hole diameter was calculated by taking the average of the longest and the corresponding perpendicular measurement. Wound size was calculated based on the formula *A *= (*d*/2)^2 ^(π), where *A *is the area of the wound in square millimeters and *d *is the mean ear-hole diameter.

### Cytokine and chemokine measurements

Quantification of murine IL-1α, IL-1β, IL-2, IL-3, IL-4, IL-5, IL-6, IL-7, IL-8, IL-10, IL-12(p40), IL-12(p70), IL-13, IL-15, IP-10, Eotaxin, IFN-γ, GM-CSF, MCP-1, MIP-1α, RANTES, TNFα and MIP-2α in mouse serum was determined in duplicative measurements using Luminex-100 technology (Austin, TX) with Fluorokine MAP Multiplex Mouse Cytokine Panels (R&D Systems, Minneapolis, MN) and Mouse Proinflammatory (7-plex) Arrays (Mesoscale Discovery, Gaithersburg, MD) in accordance to manufacturer's instructions. Sample concentrations were interpolated from standard curves, and results were expressed in pg/ml.

### RNA extraction

Total RNA was extracted from skin, lung and ear tissue and stored in RNALater (Ambion, Austin, TX). Briefly, tissue was homogenized using Trizol reagent (Invitrogen, Carlsbad, CA) and total RNA was isolated using Qiagen RNeasy Lipid Tissue Mini Kit (QIAGEN Inc. Valencia, CA) according to manufacturer's instructions. Sample quantity, and quality was assessed by determining the A_260/280, _A_260/230 _ratio on a Nanodrop 2000 Spectrophotometer (NanoDrop Technologies Inc. Wilmington, DE) and by measuring 28S/18S ribosomal RNA ratios and RNA Integrity Number (RIN) using an Agilent 2100 BioAnalyzer (Agilent Technologies Inc., Santa Clara, CA). Agilent RNA integrity values for all sampled wound specimens in this study were ≥ 8.5. Reverse transcription was performed with Roche 1^st ^Strand Synthesis kit (Roche Diagnostics Corporation, Indianapolis, IN). Briefly, 1.0 μg sample RNA was added to a master mix containing 1x reaction buffer, 5 mM MgCl_2_, 1 mM deoxynucleotide mix, 6.4 μg random primers, 100 units RNase inhibitor, and 40 units AMV reverse transcriptase. 10 mM Tris buffer, pH 7.5 was used to reach 40 μl final reaction volume. Then, final reaction mixture was subjected to a single reverse-transcription cycle of 25°C for 10 min, 42°C for 60 min, 99°C for 5 min, and 4°C for at least 10 min.

### Real-Time quantitative PCR (RT-PCR) gene profiling for pro-inflammatory transcripts

Quantitative real-time polymerase chain reaction (RT-PCR) was performed using the ABI Prism 7900 HT Sequence Detection System (Applied Biosystems, Foster City, CA). Custom designed 'Wound Repair' TaqMan^® ^Low Density Array (TLDA) cards (Applied Biosystems, Foster City, CA) and catalogued "Primer Assays" (SABiosciences, Rockville, MD) were used to assess gene expression of a small cohort of pro-inflammatory and/or anti-inflammatory cytokines: (IL-1α, IL-1β, IL-6, TNFα, MCP-1, MIP-2α, IL-10, PGE-2, MIP-1α, MIP-1β, PF-4, ENA-78, IP-10, I-TAC, and iNOS). Amplification parameters were as follows: one cycle of 50°C for 2 min and 95°C for 10 min followed by 40 cycles of 95°C for 30 sec and 60°C for 1 min.

### RT-PCR data analysis

RT-PCR data were analyzed using the Sequence Detection System version 2.3 included with the ABI Prism 7900 HT RT-PCR system or using Microsoft Excel. The threshold cycle (C_t_) for each sample was manually set to 0.2 and the baseline was set between 3 and 15 cycles. 18 S ribosomal RNA was used as an endogenous housekeeping control for normalization and the comparative C_t _method was used to calculate the relative fold expression by . Assays with C_t _values greater than 35 cycles were excluded from analysis [27,28].

### Statistics

Statistical analysis of variance was used to analyze the data and a nonparametric Mann-Whitney *U *test was used to determine the level of significance of differences in sample means (GraphPad PRISM 4.0). A p value < 0.05 was considered significant.

## Results

### Impaired ear-hole wound closure in the MRL/MpJ mouse following a severe thermal injury at a remote injury site

**Figure 1:**
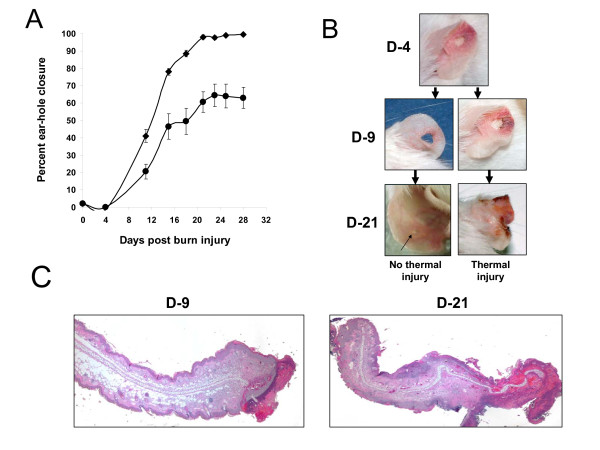
**Severe thermal injury at a remote distal site attenuates ear-hole wound closure and tissue regeneration in MRL/MpJ mice**. (A) Time course of ear-hole closure in thermally injured and solely ear-hole wounded MRL/MpJ mice. Data represent mean percent ear-hole closure ± 1 SD of ten animals in each group. (B) Macroscopic changes in ear-hole healing control and thermally injured mice. Representative photographs of the ear-hole wound sites at the indicated time post injury. The arrow points to a ear-hole wound that is 85% closed. (C) Representative histological sections of ear wound margin tissue 9 and 21 days after full-thickness third degree thermal injury (original magnification, × 40)

Full-thickness ear-hole biopsies were made in the pinnae of both ears within 1 hr after burn wounds (~177 mm^2^) were created on the mid-dorsum of each mouse. As shown in Figure 1A, ear-hole closure was significantly impaired in thermally injured mice. The average wound closure response was delayed and reduced to 65% of that of sham-treated mice. By 1-4 days post wounding, the wound edge and adjacent tissue surrounding the ear-holes became transiently inflamed (observed macroscopic reddening) in the majority of the mice (Figure 1B). Moreover, in 50-60% of the thermally injured mice the inflammatory response gradually became more progressive resulting in an augmented inflammatory cell infiltration (data not shown), which lead to extensive necrosis and fibrosis development (Figure 1B-C). Within 10-21 days wounding a large percentage (38%) had to be euthanized early during the study due to extensive necrosis, inflammation and ear deformation. The wound closure measurements for these mice were not included in the results depicted in Figure 1A. In contrast, no such ear lesions were observed in mice that were sham-injured or burned only (no ear wounds).

### Cytokine and chemokine mRNA expression in wound margin tissue from full-thickness burns

**Figure 2:**
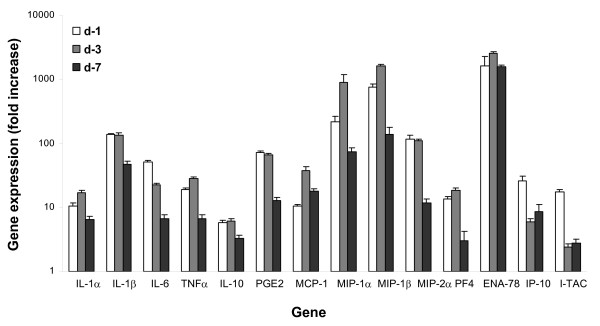
**Cutaneous dermal burn injury induces a significant local inflammatory response as evidenced by soluble mediator production**. Chemokine, proinflammatory cytokine and PGE_2 _synthase gene transcript levels at the wound edge/margin were assessed using quantitative RT-PCR as described in the *Material and Methods*. Representative results from two independents experiments are shown. The expression of transcripts normalized to 18 s at the wound margin at days 1, 3 and 7 post thermal injury were determined. Each value represents the mean ± SEM fold increase in gene expression versus naïve uninjured skin from control MRL/MpJ mice. (*n *= 10 mice/time point, all data points have P values < 0.05 as compared with naïve skin).

mRNA transcript expression levels were determined by RT-PCR and normalized to gene transcript levels in normal uninjured naïve skin (Figure 2). The expression of mRNA transcripts for IL-1α, IL-1β, TNFα, PGE_2_, MCP-1 (CCL2), MIP-1α (CCL3), MIP-1β (CCL4), MIP-2α (CXCL2), PF4 (CXCL4), and ENA-78 (CXCL5) was significantly increased at days 1 and 3 post thermal injury. By contrast, IL-6, IP-10 (CXCL10) and I-TAC (CXCL11) expression peaked at day 1 post injury and then gradually declined over the subsequent 6 days. Compared with burn wounds on Balb/c mice (data not shown), MRL/MpJ burn wounds had increased and prolonged transcription of IL-6, IL-1β and decreased transcription of IL-10 and chemokines MIP-2α, ENA-78, IP-10 and I-TAC in burn wound margin tissue [26].

### Severe thermal injury induces a systemic inflammatory response which profoundly augments the local inflammatory wound healing response as evidenced by increased soluble mediator production in the serum

**Figure 3:**
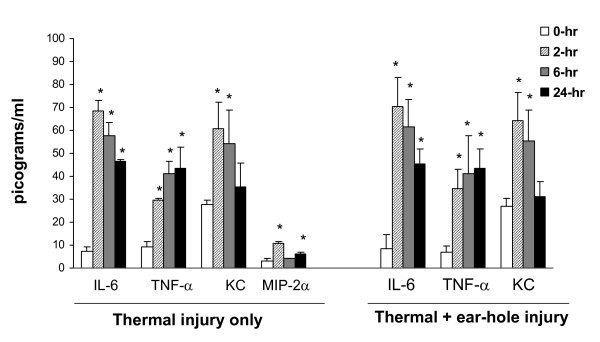
**Serum IL-6, TNFα, KC, and MIP-2α levels at 0, 2, 6 and 24 hr post thermal injury and post thermal injury plus ear punch**. Thermal injury induced a significant systemic inflammatory response, as did the dual-injury model. Serum levels were determined as described in the *Material and Methods *section. MIP-2α measurement not performed for thermal injury plus ear punch group. Data are mean ± SEM (*n *= 5-7 mice/time point, * P < 0.05 as compared with baseline time 0-hr levels)

At 2, 6 and 24 hours post burn injury, the levels of various inflammatory mediators were quantified in the serum. Four cytokines/chemokines (IL-6, TNFα, KC (CXCL1), and MIP-2α (CXCL2)) were significantly elevated within the first 24 hr post wounding compared with unburned (sham-treated) mice (Figure 3). A similar pattern was observed in dually injured (ear punch and burn) mice. IL-1β, and IL-10 were not detectable in thermally injured mice at any of the studied time points, (data not shown) and measurement of MIP-2α levels was not performed in the dually-injured group.

### Severe cutaneous thermal injury induces systemic pro-inflammatory/anti-inflammatory mediator expression in tissues remote from the wound site

**Figure 4:**
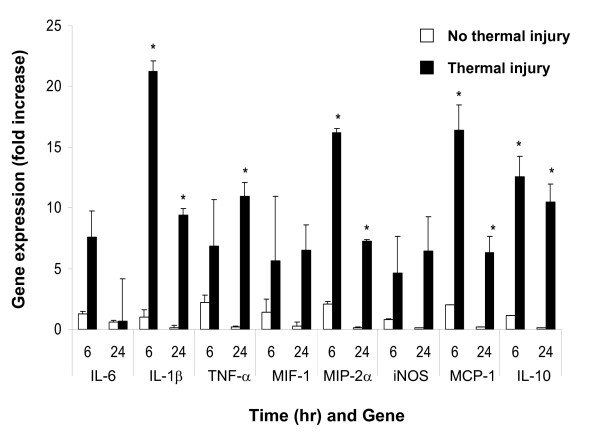
**Systemic inflammation in the lung following a local full-thickness cutaneous thermal injury**. Lungs from MRL/MpJ mice with ear-hole wounds ± thermal injury were evaluated for inflammatory gene expression. Severe thermal trauma induced significant elevation of lung inflammatory chemokine, and iNOS transcript levels at 6 and 24 hr post injury in the lung. Gene levels were assessed using quantitative RT-PCR as described in the *Material and Methods*. The expression of transcripts normalized to 18 s in the lung at 6 and 24 hrs post thermal injury were determined. Each value represents the mean ± SEM fold increase in gene expression versus lung tissue from uninjured control MRL/MpJ mice (*n *= 5-7 mice/time point, * P < 0.05 as compared with baseline time 0-hr levels)

**Figure 5:**
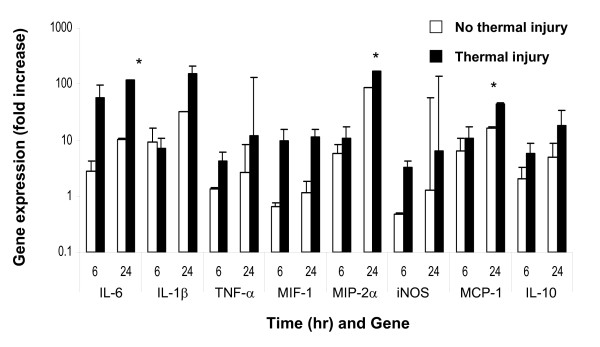
**Severe local thermal injury markedly heightens the remote ear-hole inflammatory processes resulting in attenuated tissue healing**. MRL/MpJ mice with ear-hole wounds ± thermal injury were evaluated. Gene transcript levels obtained from tissue collected at the edge/margin of the ear-hole wounds were assessed using quantitative RT-PCR as described in the *Material and Methods*. The expression of transcripts normalized to 18 s in ear tissue at 6 and 24 hrs post thermal injury were determined. Each value represents the mean ± SEM versus ear tissue from uninjured control MRL/MpJ mice (*n *= 5-7 mice/time point, * P < 0.05 as compared with baseline time 0-hr levels).

Significantly upregulated expression of mRNA transcripts for proinflammatory cytokine/chemokines IL-6, IL-1β, TNFα, MIF-1α, MIP-2α and MCP-1 as well as the anti-inflammatory cytokine IL-10 and inducible nitric oxide synthase (*i*NOS) were measured in the wounded lung and ear at 6 and 24 hrs post thermal injury (Figures 4 and 5). Marked inflammation, as evidenced by the gross and histological appearance of the wound (Figures 1B-C) and increased global expression of potent inflammatory mediators (Figure 5), was seen in the early ear wound healing in the thermally injured mouse.

## Discussion

Numerous studies have shown that severe traumatic injury can lead to systemic pro-inflammatory responses and cellular immune dysfunction [4-8,12,13,16]. In this study, we demonstrate that systemic wound trauma inflammatory signaling, mediated by acute thermal injury, attenuates normal ear-hole closure and scarless healing in MRL/MpJ mice. Based on our previous findings that MRL/MpJ mice exhibit a delay in wound closure and myofibroblast development following sever thermal trauma, and that these mice show a propensity to develop the autoimmune state lupus following significant tissue injury [22,26], we hypothesized that the activation of specific cell types and the production of cytokines and other wound healing reparative mediators may be detrimental to a remote ear-hole tissue regenerative response in MRL/MpJ mice following traumatic tissue injury. Several other groups have examined the link between local injury and a systemic response. Similar to our findings Schwacha et al [29], reported a significant inflammatory response after burn wounds in small animals and delayed wound healing distant to the site of injury.

In the present study, we show attenuated ear-hole closure and tissue regeneration in a large percentage of MRL/MpJ mice following a remote, severe, full-thickness, cutaneous thermal injury. Pathological examinations of ear-hole wounds demonstrated excessive sequestration and infiltration of macrophage and neutrophils. The majority of the MRL/MpJ ear wounds healed with histological evidence of fibrosis and scar formation or became chronically inflamed and necrotic to the point where some of the animals had to be euthanized. The precise reasons for aberrant ear-hole healing (wound closure) in these mice following thermal injury are unclear, but may be related to heightened systemic inflammation, higher fibrotic cytokine signaling and the overproduction of danger signals (presence of pathogens, pathogen-derived molecules, or even self-derived molecular danger signals, which arise from tissue damage) [30] which lead to uncharacteristic healing and scar formation.

An essential feature of scarless healing/tissue regeneration in adults and in fetal tissues appears to be a diminished cytokine response to injury [1,31,32]. On the other hand, cytokines introduced into the fetal environment evoke heightened inflammatory responses and tissue fibrosis [33,34]. Similarly, PGE_2 _stimulates leukocyte accumulation, fibroblast proliferation, and collagen deposition resulting in delayed wound healing and scar formation when introduced into early fetal wounds [35].

Elevated levels of IL1-β, IL-6, TNFα, PGE_2_, iNOS and various chemokines are associated with areas of local as well as systemic inflammation [13]. We observed a heightened inflammatory response at the site of a remote secondary injury following severe burn trauma; a pathological picture suggestive of a global systemic immune response. In contrast, blunted expression and production of IL-1β, IL-6, TNFα, iNOS correlated with complete ear-hole closure and scarless tissue healing. Pathological examinations of ear-hole wounds in thermally injured mice demonstrated excessive sequestration and infiltration of macrophage and neutrophils. These observations lead us to speculate that macrophage hyperactivity after thermal injury may play a critical role in altered ear-hole healing response, as primed hyperactive macrophages might contribute to the increased recruitment and sequestration of leukocytes, tissue inflammation, and damage-tissue necrosis precipitated through excessive cytokine and chemokine production [36].

## Conclusion

Collectively, the findings from this study have important implications in defining how immune responses induced following a severe traumatic injury regulate other cellular events critical in distal wound healing. Although the mechanism(s) mediating scarless healing are not fully understood, an absence of local as well as systemic inflammation seems to be particularly important for scarless healing in the MRL/MpJ mouse [21,23-25]. We speculate that optimal ear-hole healing requires activation of inhibitory signals that suppress chemokine and cytokine synthesis, resulting in resolution of the inflammatory infiltrate. The effects of trauma on healing and tissue regeneration responses may depend upon the stimuli for its induction, its anatomical site, the degree of immune cell activation and the time post burn at which the injury is induced. Our findings are not altogether surprising; extensive tissue damage can trigger potent cellular activation signals that prime innate immune system to sense "danger" [30,37] and that tissue regeneration and scarring are tightly regulated by inflammation. Additional studies are warranted and we believe that this is an appropriate model to investigate the pathomechanisms of normal and aberrant wound healing.

## List of abbreviations used

CCL-: chemokine ligand with cysteine-cysteine motif; CXC-: chemokine with cysteine-amino acid-cysteine motif; ENA-78: epithelial cell-derived neutrophil-activating peptide-78; GM-CSF: granulocyte/macrophage colony stimulating factor; IFN-γ: interferon gamma; IL- interleukin-; iNOS: inducible nitric oxide synthase; IP-10: interferon inducible protein 10; I-TAC: interferon inducible t cell alpha; KC: cytokine induced neutrophil chemoattractant; MCP-1: monocyte chemoattractive protein-1; MIP-: macrophage inflammatory protein-; NMRC: Naval Medical Research Center; PF-4: platelet factor 4; PGE_2_: prostaglandin E2; RANTES: regulated upon activation, normal T cell expressed and secreted; RT-PCR: real-time polymerase chain reaction; TGF-: transforming growth factor-; TNF-: tumor Necrosis Factor-; USUHS: Uniformed Services University of the Health Sciences; WRAIR: Walter Reed Army Institute of Research.

## Competing interests

The authors declare that they have no competing interests.

## Authors' contributions

TAD conceived and designed the research. TAD, MFA, KA and SRZ carried out all the experimental work and data collection. TAD, MFA, KA, EAE and SRZ conducted the data analysis and interpretation. TAD, EAE, and SRZ wrote the manuscript and/or made critical revisions. All authors read and approved the final version of the manuscript.

## About the Authors

The authors are employees of the U.S. Government. This work was prepared as part of their official duties. Title 17 U.S.C. *§*105 provides that 'Copyright protection under this title is not available for any work of the United States Government.' Title 17 U.S.C *§*101 defines a U.S. Government work as a work prepared by a military service member or employees of the U.S. Government as part of that person's official duties. The opinions or assertions contained in this paper are the private views of the authors and are not to be construed as reflecting the views, policy or positions of the Department of the Navy, Department of Defense nor the U.S. Government.
